# Current knowledge on exocrine glands in carabid beetles: structure, function and chemical compounds

**DOI:** 10.3897/zookeys.100.1527

**Published:** 2011-05-20

**Authors:** Anita Giglio, Pietro Brandmayr, Federica Talarico, Tullia Zetto Brandmayr

**Affiliations:** Department of Ecology, University of Calabria, via P. Bucci, I-87036 Arcavacata di Rende (CS), Italy

**Keywords:** antennal glands, pygidial gland, defensive secretion, carabid beetles

## Abstract

Many exocrine products used by ground beetles are pheromones and allomones that regulate intra- and interspecific interactions and contribute to their success in terrestrial ecosystems. This mini-review attempts to unify major themes related to the exocrine glands of carabid beetles. Here we report on both glandular structures and the role of secretions in carabid adults, and that little information is available on the ecological significance of glandular secretions in pre-imaginal stages.

## Introduction

Exocrine gland secretions in insects are involved in reproductive and defensive behaviour (Pasteels et al. 1983; Blum 1996), and are important in social integration and communication among members of the same colony (as in Hymenoptera) (Hölldobler and Wilson 1990). These exocrine glands have an ectodermal origin and morphological or functional classifications have been generally used to describe them. The location and morphology of these glands are directly related to their function (Billen 1998). Many glands are common to all insects, e.g. mandibular and salivary glands, male and female accessory glands associated with reproductive organs (Dallai et al. 1999; Viscuso et al. 2001) and defensive glands (Thiele 1977), whereas some glands are characteristic of a family or species (Grassé 1975; Quennedey 1998, 2000), especially in social insects (Cammaerts 1974; Bin et al. 1989; Hölldobler and Wilson 1990, also see reviews; Billen 1991; Delfino et al. 1991, 1992; Pedata et al. 1993; Bartlet et al. 1994; Isidoro and Bin 1995; Isidoro et al. 1996, 2000; Bot et al. 2001; Gobin et al. 2001, 2003; Torres et al. 2001).

Information on the chemistry of defensive secretions in many carabid species are available in Dettner (1987), Whitman et al. (1990) and Will et al. (2000). In this manuscript, carabid beetles are meant in the widest sense of the word, including the old lineage of Trachypachidae, the Rhysodidae and the Paussinae as a subfamily (as in Beutel and Leschen 2005). The nomenclature of palaearctic taxa follows Löbl and Smetana (2003).

## Adult antennal glands

The cellular architecture of adult antennal glands has been investigated for *Platynus assimilis* (Paykull 1790) (Weis et al. 1999), *Paussus* spp Linnaeus 1775 (Di Giulio et al. 2003, 2009; Nagel 1979) and *Siagona europaea* Dejean 1826 (Giglio et al. 2005). Structural analysis shows a great number of antennal glands that have been classified into the following main categories (Noirot and Quennedey 1991, Quennedey 1998): i) unicellular gland class 2, which is not in contact with the cuticle; ii) bi- and tri-cellular gland class 3, connected to the cuticle by a cuticular duct draining the secretions outside. The first type (class 2) includes unicellular glands known as oenocytes. They are located only within the antennal lumen of *Siagona europaea* and are not found in other carabid species (Giglio et al. 2005). Their role in cuticular hydrocarbons secretions is suggested by Lockey (1988) and Noirot and Quennedey (1991). The second type are tri-cellular glands, composed of a secretory, an intercalary and a duct cell, and are found in *Paussus assimilis* (Weis et al. 1999), *Paussus favieri* Fairmaire (Di Giulio et al. 2009)and *Siagona europaea*. Moreover, a large number of bi-cellular glands, composed of one gland and one duct cell, are located on the antennal surface of *Paussus favieri*. The structural variability and distribution of the antennal glandular apparatus on Paussini, such as the myrmecophilous *Paussus favieri*, are closely related to their symbiotic life style (Geiselhardt et al. 2007). Predators, such as *Paussus assimilis* and *Siagona europaea*, which have free-living life habits, show a more simple glandular apparatus. Exocrine gland class 3 of the myrmecophagous *Siagona europaea* produces secretions that protect the surface of the antennae and sensilla from wear.

## Pygidial glands

Ground beetles possess a pair of abdominal glands known as pygidial glands, which produce defensive secretions. Their structure consists of two sets of secretory lobes, collecting canals, collecting reservoirs and has been well described for many species (Benn 1973; Forsyth 1970, 1972; Scott et al. 1975; Balestrazzi et al. 1985; Rossini et al. 1997; Eisner et al. 2000; Will et al. 2000, 2010; Attygalle et al. 2004). These glands are variable in structure and in the nature of the produced substances (Thiele 1977), and discharge the secretion products by oozing, spraying or crepitation. Oozing is probably the plesiotypic mode of discharge, with active spraying and crepitation as later refinements (Moore 1979). The main function of pygidial glands is probably in the defence against predators, but also in the facilitation of the penetration of defensive compounds into the predator’s integuments, antimicrobial and antifungal activity, and in producing alarm messages (Evans and Schmidt 1990; Blum 1996).

A comparative study of the secretions of carabid pygidial glands was made by Schildknecht et al. (1968). Moore (1979) and Will et al. (2000) listed all the principal groups of secretions detected in carabid tribes: hydrocarbons, aliphatic ketones, saturated esters, formic acid, higher saturated acids, unsaturated carboxylic acids, phenols (m-cresol), aromatic aldehydes (salicylaldehyde) and quinones. Attygalle et al. (1991) showed that D8-L-valine is incorporated into methacrylic and isobutyric acids in the pygidial defensiveglands of *Scarites subterraneus* Fabricius 1775. The pygidial glands of *Helluomorphoides clairvillei* (Dejean 1831) females discharge a mixture of compounds including carboxylic acid, aliphatic esters and hydrocarbons (Attygalle et al. 1992). The taxonomic distribution of defensive secretions was reviewed by Will et al. (2000) for 47 tribes. Data have shown a close relationship between chemical classes and habitat diversification. Tribes with high species diversity in tropical-subtropical and steppe habitats use formic acid as primary chemical defences, while tribes with high diversity in temperate regions use carboxylic acids, phenols, quinone, aromatic aldeydes and ketones. This can be explained by the interaction of ground beetles with their predators and prey. Specifically, ants are hypothesized to have had a major influence on the evolution of ground beetle secretions in tropical species. Bombardier beetles of the genus *Brachinus* Weber 1801 are able to release irritating quinones, produced by the oxidation of hydroquinones in a double-chambered apparatus (Schildknecht 1961; Eisner and Meinwald 1966; Schildknecht et al. 1968; Aneshansely et al. 1969; Eisner and Aneshansely 1999; Eisner et al. 2000); a certain amount of heat and the explosion associated with the reaction reinforce the defensive effect. Predation on these beetles appears to be rare (Juliano 1985; Bonacci et al. 2006, 2008). From the literature it is known that *Anchomenus dorsalis* (Pontoppidan, 1763) produces toxic methylsalycilate from its pygidial glands (Schildknecht 1970). Tiger beetle species living in moist habitats produce benzaldehyde (Altaba 1991). The carabid beetle *Galerita lecontei* Dejean 1831 secretes, as a spray, a mixture of formic acid, acetic acid and lipophilic components (long-chain hydrocarbons and esters) (Rossini et al. 1997). Biosynthesis of tiglic and ethacrylic acids from isoleucine via 2-methylbutyric acid was demonstrated in *Pterostichus californicus* (Dejean 1828) (Attygalle et al. 2007). Complex mixtures of monoterpenes are found in the defensive secretions of *Ardistomis schaumii* Leconte 1857 and *Semiardistomis puncticollis* Dejean 1831. The presence of monoterpenes in beetle secretions is well known, yet it is not very common to find the opposite enantiomers in secretions in related species (Attygalle et al. 2009).

## Exocrine glands of larval and pupal stages

Although exocrine glands and their defensive secretions are well investigated in adults, hardly any information exists for the larval and pupal stages, which are the most vulnerable stages of the beetle’s life cycle.

Glandular organs have been found in the larval stage of myrmecophilus *Pseudomorpha* sp. These glands are located on the head and thorax and secrete chemical compounds which repel ants (Erwin 1981). In Paussini larvae (*Paussus kannegieteri* Wasmann 1896) as well as in Metriini (*Metrius*) and Ozaenini, the modified terminal abdominal segments have glandular pores that secrete pleasant substances to attract their host ants (Arndt et al. 2005; Geiselhardt et al. 2007; Di Giulio 2008).

In the pupal stage of *Carabus lefebvrei* Dejean, 1826, Sturani (1962) described a “flavour humour” and suggested that this secretion has a waterproofing or an antipredatory function. Ultrastructural analyses have shown that this exudate is secreted by an acinose abdominal complex of exocrine glandular units (Giglio et al. 2009). The independent glandular unit consists of a single secretory cell, a duct and its associated cell and belongs to gland cell class 3 according to the classification of Quennedey (1998). In the cytoplasm, the secretory cell contains abundant rough endoplasmatic reticula, glycogen granules, numerous mitochondria and many well-developed Golgi complexes producing electron-dense secretory granules. Mitochondria are large, elongated and often adjoining electronlucent vesicles.Their close association with tracheoles suggests very high aerobic metabolism. Chemical analyses of the gland secretions revealed a mixture of low molecular weight terpenes as well as ketones, aldehydes, alcohols, esters and carboxylic acids, which in adults are regarded deterrents against predators. Monoterpenes, especially linalool, were the main chemical products produced by the pupal stage of *Carabus lefebvrei*. It is suggested that this gland secretion has both a deterrent function against predators and a prophylaxis function against pathogens.

## Conclusions and future studies

The present manuscript summarized the main knowledge on the exocrine glands in ground beetles. The main characteristic of glandular secretions of each life stage is its diversity and dependence on interspecific relations in the ecological niches of species. Our main future aim is to accumulate data on defensive secretions to understand, i) the mode of action of chemical compounds, and ii) species-specific variation of glandular structures and chemical secretions, paying particular attention to morphological, phylogenetic and behavioural aspects. Moreover, the need for more detailed studies on larval and pupal stages has already been stressed. Presently, the pupal stages of carabid beetles are known not to possess any physical protection, thus chemical protection provided by the abdominal glands is very important. This stage is present in environments rich in bacterial and fungal microorganisms, some of which are possible insect pathogens. Besides, the highly lipophilic nature of monoterpene compounds suggests that their principal targets are bacterial and/or fungal cell membranes.

To support this hypothesis additional research is needed to evaluate the range of activity of the secretions of pupal abdominal glands towards microorganisms and fungal entomopathogens.

## Acknowledgements

This paper is dedicated to the dear memory of Prof. Tullia Zetto Brandmayr, who enthusiastically contributed to this study until her last days.
